# Developing and Evaluating a Measure of the Willingness to Use Pandemic-Related mHealth Tools Using National Probability Samples in the United States: Quantitative Psychometric Analyses and Tests of Sociodemographic Group Differences

**DOI:** 10.2196/38298

**Published:** 2023-02-07

**Authors:** Wilson Vincent

**Affiliations:** 1 Department of Psychology and Neuroscience Temple University Philadelphia, PA United States

**Keywords:** COVID-19, psychometric properties, mHealth, digital health, digital screening, digital tracking, pandemic, national survey, mobile health, digital health tool, vulnerable population, demographic characteristic, instrument validation

## Abstract

**Background:**

There are no psychometrically validated measures of the willingness to engage in public health screening and prevention efforts, particularly mobile health (mHealth)–based tracking, that can be adapted to future crises post–COVID-19.

**Objective:**

The psychometric properties of a novel measure of the willingness to participate in pandemic-related screening and tracking, including the willingness to use pandemic-related mHealth tools, were tested.

**Methods:**

Data were from a cross-sectional, national probability survey deployed in 3 cross-sectional stages several weeks apart to adult residents of the United States (N=6475; stage 1 n=2190, 33.82%; stage 2 n=2238, 34.56%; and stage 3 n=2047, 31.62%) from the AmeriSpeak probability-based research panel covering approximately 97% of the US household population. Five items asked about the willingness to use mHealth tools for COVID-19–related screening and tracking and provide biological specimens for COVID-19 testing.

**Results:**

In the first, exploratory sample, 3 of 5 items loaded onto 1 underlying factor, the willingness to use pandemic-related mHealth tools, based on exploratory factor analysis (EFA). A 2-factor solution, including the 3-item factor, fit the data (root mean square error of approximation [RMSEA]=0.038, comparative fit index [CFI]=1.000, standardized root mean square residual [SRMR]=0.005), and the factor loadings for the 3 items ranged from 0.849 to 0.893. In the second, validation sample, the reliability of the 3-item measure was high (Cronbach α=.90), and 1 underlying factor for the 3 items was confirmed using confirmatory factor analysis (CFA): RMSEA=0, CFI=1.000, SRMR=0 (a saturated model); factor loadings ranged from 1.000 to 0.962. The factor was independently associated with COVID-19–preventive behaviors (eg, “worn a face mask”: r=0.313, SE=0.041, *P*<.001; “kept a 6-foot distance from those outside my household”: r=0.282, SE=0.050, *P*<.001) and the willingness to provide biological specimens for COVID-19 testing (ie, swab to cheek or nose: r=0.709, SE=0.017, *P*<.001; small blood draw: r=0.684, SE=0.019, *P*<.001). In the third, multiple-group sample, the measure was invariant, or measured the same thing in the same way (ie, difference in CFI [ΔCFI]<0.010 across all grouping categories), across age groups, gender, racial/ethnic groups, education levels, US geographic region, and population density (ie, rural, suburban, urban). When repeated across different samples, factor-analytic findings were essentially the same. Additionally, there were mean differences (ΔM) in the willingness to use mHealth tools across samples, mainly based on race or ethnicity and population density. For example, in SD units, suburban (ΔM=–0.30, SE=0.13, *P*=.001) and urban (ΔM=–0.42, SE=0.12, *P*<.001) adults showed less willingness to use mHealth tools than rural adults in the third sample collected on May 30-June 8, 2020, but no differences were detected in the first sample collected on April 20-26, 2020.

**Conclusions:**

Findings showed that the screener is psychometrically valid. It can also be adapted to future public health crises. Racial and ethnic minority adults showed a greater willingness to use mHealth tools than White adults. Rural adults showed more mHealth willingness than suburban and urban adults. Findings have implications for public health screening and tracking and understanding digital health inequities, including lack of uptake.

## Introduction

Public health responses to COVID-19 and other pandemics (eg, SARS outbreaks, Zika virus disease, swine influenza, the 1918 influenza pandemic) require the public’s strong willingness to participate in preventive and screening efforts [1,2]. As screening advances develop and deploy in response to outbreaks and pandemics, at-home and mobile health (mHealth) methods could ease the burden on testing infrastructure, such as supply chain issues, and limit the public's exposure to pathogens [3,4]. At-home and mHealth strategies successful for HIV screening were deployed in response to COVID-19 [3,5]. However, current events and recent studies have revealed significant variability among the public's participation in screening and preventive measures to address COVID-19, including vaccinations or even mask wearing or social distancing [6,7]. Additionally, the uptake of COVID-19–related mobile apps has been relatively modest worldwide [8], and acceptance of contact-tracing apps was low in most countries [9]. We lack psychometrically validated, rigorously tested measures to help understand people’s willingness to engage in screening and prevention efforts, such as digital tracking, or screen participants for mHealth and pandemic-related research.

A review of the peer-reviewed COVID-19 literature found that mHealth solutions are used for many different aims, including early detection, rapid screening, patient monitoring, and treatment [10]. The most commonly used modalities were mobile phone–based apps and SMS (ie, text messaging) [10]. Most mobile apps in the early months of the pandemic were focused on contact tracing, with other apps focused on symptom monitoring and educational or informational content [11,12]. These included downloadable apps in the Android Play Store and the iOS App Store [11]. Contact-tracing apps, in particular, grew rapidly within the first few months of the COVID-19 pandemic in the United States [8]. A general measure of the willingness to use mHealth tools for public health screening and tracking and information-seeking purposes can help researchers and practitioners identify the correlates, predictors, and outcomes of using such mHealth apps and screen for and evaluate mHealth interventions.

This paper found no published examples of psychometrically validated scales for assessing the willingness to use mHealth-related tools for public health purposes. Studies have typically focused on 1 question or a few separate questions to assess the willingness to use such tools. For example, in an extensive survey of registered National Health Service users in the United Kingdom, participants responded “yes,” “no,” or “unsure” to 1 question asking about their willingness to participate in mHealth app–based contact tracing [13]. The use of 1 question to assess the willingness to use mHealth tools in the study indicates the need for measures to be brief if they attempt to be any more comprehensive.

Other studies have included several items about the intentions or willingness to use mobile apps in general and, alternatively, specific apps that the researchers were evaluating. For example, a previous study used a 3-item measure of the general intention to use medical apps [14]. The items were not specific about the nature of the medical mHealth apps, such as whether they would be used for contact tracing, symptom tracking, or provision of information about an illness [14]. Items that include examples of various uses for medical mHealth apps (eg, contact tracing, symptom tracking, or detection) could help us account for participants’ limits in using these apps; for example, a participant who would use mHealth tools for educational purposes would not necessarily want to use them for contact tracing or symptom tracking. Another study asked participants one question about how willing they would be to install an app for contact tracing and another question about how willing they would be to keep an app that was automatically downloaded to their smartphone [15]. An additional study used a 2-item measure of behavioral intentions to use digital health technologies for COVID-19 as a proxy of actual use, although the authors acknowledged that behavioral intentions and actual use are separate constructs [16]. A different study used a single-item measure of willingness or support for using a specific contact-tracing app being evaluated by the researchers, and participants’ responses were categorized as “app-supporting,” “app-willing,” and “app-reluctant” [17]. Finally, a study asked 3 questions (eg, plan to use the app, hope the app becomes available for use) so that participants could rate their intention to use 2 specific mobile apps that the researchers were evaluating, one for contact tracing and the other for symptom tracking [18]. None of these studies assessed the extent to which the items they used jointly reflected a single underlying willingness to use mHealth tools.

A critical reason a broadly applicable measure of the willingness to use mHealth tools is needed is that it can help researchers better understand the inequities in mHealth uptake and outcomes, including sociodemographic factors related to digital health inequities [19]. There are barriers to mHealth that suggest a digital divide that adversely affects some demographic groups compared to others. For example, although a large survey of registered National Health Service users in the United Kingdom found no differences by age or gender in terms of the willingness to participate in contact tracing via a mobile app [13], other studies have found different results. For instance, older age [17,18] and higher socioeconomic status (ie, lower financial deprivation) [17] have been associated with a greater willingness or intention to use specific mobile apps. Further, studies have found that older patients are less likely to use telehealth, broadly speaking, not just mHealth tools [20], and older patients living in a rural area counted on the United States-Mexico border reported less satisfaction with telehealth compared to younger adults living in the same area [21]. In addition, urban patients have shown greater willingness to use telehealth than rural patients [20]. However, the aforementioned rural patients in the study conducted at the United States-Mexico border showed high levels of satisfaction with telehealth and expressed a willingness to use telehealth in the future despite within-group age differences [21]. Additionally, a small study of patients with comorbid depression and diabetes who receive public health care services in San Francisco found that these patients of lower socioeconomic status had high interest in using digital platforms to manage their health after the onset of the COVID-19 pandemic [22]. However, the authors also found that these patients may require additional human support to help them gain access and get started with being able to use the technology [22]. Approximately a third of these patients reported needing assistance with using their smartphone and installing apps [22].

Researchers and mHealth thought leaders have asserted that it will be helpful to provide and increase access to digital health solutions along with education regarding digital health [19,23]. Additionally, digital health equity should be centered within digital health efforts [19], including those focused on pandemics, such as COVID-19. To these ends, it may be important to measure the willingness to use such technologies to understand what educational, supportive, or motivational efforts are needed to facilitate uptake among populations adversely affected by digital health inequities.

The 2020 COVID Impact Survey (CIS) [24] was a national probability household survey conducted by the National Opinion Research Center (NORC) at the University of Chicago to estimate the impact of COVID-19 on the United States. The CIS used 5 items to assess the willingness to use mHealth tools for COVID-19–related screening and tracking (3 items) and the willingness to test for COVID-19 by providing biological specimens (2 items). Multiple studies have used these CIS items, which could easily be used or adapted for future pandemics or outbreaks. However, studies have used each item individually, without considering them as a single scale. Studies have yet to assess the psychometric properties of the measure in a comprehensive manner.

Given that there are no psychometrically validated measures to screen for the willingness to participate in pandemic-related screening and tracking, including the willingness to use mHealth tools for screening and tracking, this study aims to validate such a measure in a large, national probability sample. Using data from the CIS, the following were assessed: one-dimensionality versus multidimensionality (eg, whether the measure assesses a single construct representing the willingness to participate in any screening and tracking or multiple constructs), convergent and discriminant validity based on its associations with expected correlates of willingness for screening and tracking, measurement invariance (ie, whether the measure assesses the same construct in the same way across sociodemographic or cultural groups, specifically age groups, gender, racial or ethnic groups, education level, geographic regions of the United States in which adults live, and population density of adults’ lived community [ie, rural, suburban, urban]), and mean differences in the underlying factor across demographic and cultural groups.

## Methods

### Data

Data from all 3 cross-sectional stages of the CIS conducted in 2020 were used: N=6475; stage 1 (April 20-26), n=2190, 33.82%; stage 2 (May 4-10), n=2238, 34.56%; and stage 3 (May 30-June 8), n=2047, 31.62%. Given the cross-sectional nature of the data collection, households were not tracked for repeated assessment across the 3 stages. The data were collected using the AmeriSpeak Panel, a probability-based panel implemented by NORC at the University of Chicago, covering approximately 97% of the US household population. The CIS sampled US households with a known, nonzero probability of selection based on the NORC National Sample Frame, which was extracted from the US Postal Service Delivery Sequence File. Households were contacted by US mail, email, telephone, and field interviewers. The data represent noninstitutionalized adults who reside in the United States when weighted using sampling weights provided by the CIS.

Fundamentally, the process of selecting households for the CIS was based on random selection within a sampling frame of oversampling to account for expected, differential rates of survey completion or population coverage for different demographic groups (eg, younger adults, racial and ethnic minority groups) and geographical areas. The prospective households were stratified by geographic region along with age, gender, race or Hispanic ethnicity, and education level. Within these stratifications, households were randomly selected. The differential probabilities of selection and response based on demographic characteristics and geographic region were used to construct the sampling weights that were accounted for in these analyses. The only criteria beyond efforts to adequately represent the population based on the probability of selection and expected survey completion rates were that there was an adult in the household who could complete the survey in English or Spanish either online or via telephone. Detailed reports of all methods of the CIS, including household selection, are available online for the stage 1 [25], stage 2 [26], and stage 3 [27] samples.

### Ethical Considerations

The NORC Institutional Review Board reviewed and approved the CIS study protocol for the protection of human subjects’ rights and welfare (FWA00000142). The CIS adhered to all federal and local guidelines and regulations. All subjects who participated in the CIS data collection provided informed consent and were informed that their identities would remain confidential. The original informed consent allows for secondary data analysis, as in this study, provided that the data are deidentified. In fact, the data are deidentified. For example, the data producer, NORC at the University of Chicago, omitted the true stratum and cluster variables from these complex survey data to preserve confidentiality. The data producer found that the cluster variable was negligible, and provided an appropriate pseudostratum variable to be used in place of the true variable. The Temple University Institutional Review Board determined that secondary analyses of deidentified data, such as this study, do not constitute human subject research and, thus, do not require review or approval.

### Measures

The following CIS measures in the present analyses were used: (1) willingness to participate in pandemic-related screening and tracking, (2) correlates of the willingness to participate in pandemic-related screening and tracking, and (3) sociodemographic characteristics.

#### Willingness to Participate in Pandemic-Related Screening and Tracking

Participants responded to questions asking about their likelihood of providing biological specimens for COVID-19–related testing (ie, “testing you for COVID-19 infection using a Q-tip to swab your cheek or nose,” “testing you for immunity or resistance to COVID-19 by drawing a small amount of blood”) and digital screening and tracking (eg, “installing an app on your phone that asks you questions about your own symptoms and provides recommendations about COVID-19,” “installing an app on your phone that tracks your location and sends push notifications if you might have been exposed to COVID-19”; see Tables 1 and 2). Response options ranged from “1. Extremely likely” to “5. “Not at all likely.” Items were reverse-coded such that higher scores reflected a greater perceived likelihood for screening and tracking. Participants had the option to respond with “88. Already done this,” and these cases were excluded from primary analyses using listwise deletion.

**Table 1:** Demographic characteristics and descriptive statistics at stage 1 (N=2190)^a^.

Variable			Descriptive statistics						
			Participants, n (%)		Estimated proportion		Linearized SE		Design effect
**Age (years)**									
	18-29	282 (12.87)		0.205		0.016		3.21	
	30-44	672 (30.68)		0.254		0.013		1.92	
	45-59	524 (23.93)		0.243		0.013		2.13	
	≥60	712 (32.51)		0.298		0.014		2.07	
**Gender**									
	Male	1036 (47.31)		0.483		0.016		2.25	
	Female	1154 (52.69)		0.517		0.016		2.25	
**Race/ethnicity**									
	Asian/Asian American	48 (2.19)		0.031		0.006		2.93	
	Black/African American	267 (12.19)		0.119		0.010		2.14	
	Hispanic/Latinx	369 (16.85)		0.167		0.013		2.78	
	White/European American	1397 (63.79)		0.628		0.016		2.35	
	Other races and ethnicities	109 (4.98)		0.055		0.007		2.27	
**Education level**									
	No high school diploma	98 (4.47)		0.098		0.012		3.67	
	High school diploma or equivalent	405 (18.49)		0.283		0.016		2.68	
	Some college	893 (40.78)		0.277		0.012		1.67	
	Bachelor’s degree or above	794 (36.26)		0.343		0.015		2.09	
**Geographical region of the United States**									
	Northeast	323 (14.75)		0.174		0.010		1.41	
	Midwest	547 (24.98)		0.207		0.009		1.10	
	South	770 (35.16)		0.380		0.012		1.25	
	West	550 (25.11)		0.238		0.010		1.15	
**Population density of one’s lived community**									
	Rural	155 (7.08)		0.095		0.009		2.21	
	Suburban	406 (18.54)		0.201		0.013		2.14	
	Urban	1624 (74.16)		0.703		0.014		2.14	

^a^Percentages may not sum up to 100 due to rounding. Subcategories may not sum up to 2190 due to missing data.

**Table 2:** Descriptive statistics at stage 1 (N=2190)^a^.

Variable			Descriptive statistics						
			Participants, n (%)		Estimated proportion		Linearized SE		Design effect
**Which of the following measures, if any, are you taking in response to the coronavirus? (Answered yes)**									
	Canceled a doctor appointment	739 (33.74)		0.324		0.015		2.20	
	Worn a face mask	1713 (78.22)		0.775		0.014		2.37	
	Visited a doctor or hospital	167 (7.63)		0.079		0.009		2.44	
	Canceled or postponed work activities	704 (32.15)		0.324		0.015		2.26	
	Canceled or postponed school activities	448 (20.46)		0.212		0.013		2.33	
	Canceled or postponed dentist or other appointment	819 (37.40)		0.360		0.015		2.15	
	Canceled outside housekeepers or caregivers	221 (10.09)		0.114		0.011		2.70	
	Avoided some or all restaurants	1574 (71.87)		0.716		0.015		2.25	
	Worked from home	758 (34.61)		0.322		0.015		2.11	
	Studied at home	325 (14.84)		0.148		0.012		2.41	
	Canceled or postponed pleasure, social, or recreational activities	1554 (70.96)		0.692		0.015		2.37	
	Stockpiled food or water	765 (34.93)		0.325		0.015		2.14	
	Avoided public or crowded places	1762 (80.46)		0.805		0.013		2.24	
	Prayed	1212 (55.34)		0.560		0.016		2.24	
	Avoided contact with high-risk people	1384 (63.20)		0.621		0.016		2.30	
	Washed or sanitized hands	2037 (93.01)		0.918		0.010		3.15	
	Kept a 6-foot distance from those outside my household/your household	1913 (87.35)		0.855		0.012		2.67	
	Stayed home because I felt unwell/you felt unwell	252 (11.51)		0.106		0.010		2.25	
	Wiped packages entering my home/your home	998 (45.57)		0.453		0.016		2.25	
**[After COVID-19 began spreading in the United States,] in the past month, how often did you communicate with friends and family by phone, text, email, app, or the internet?**									
	Basically every day	1434 (65.48)		0.648		0.015		2.28	
	A few times a week	544 (25.30)		0.244		0.014		2.17	
	A few times a month	133 (6.07)		0.067		0.009		2.92	
	Once a month	45 (2.05)		0.027		0.005		2.45	
	Not at all	19 (0.87)		0.006		0.002		1.31	
**During a typical month prior to March 1, 2020, when COVID-19 began spreading in the United States, how often did you communicate with friends and family by phone, text, email, app, or the internet?**									
	Basically every day	1177 (53.74)		0.541		0.016		2.26	
	A few times a week	740 (33.79)		0.324		0.015		2.25	
	A few times a month	203 (9.27)		0.094		0.009		2.05	
	Once a month	43 (1.96)		0.026		0.006		3.50	
	Not at all	13 (0.59)		0.007		0.003		3.11	
**Willingness to get digitally tracked and tested for COVID-19 (If these options were available to you, how likely would you be to participate in them?): installing an app on your phone that asks you questions about your own symptoms and provides recommendations about COVID-19**									
	Extremely likely	290 (13.24)		0.134		0.011		2.44	
	Very likely	308 (14.06)		0.140		0.011		2.32	
	Moderately likely	437 (19.95)		0.208		0.014		2.52	
	Not too likely	441 (20.14)		0.196		0.012		2.14	
	Not likely at all	678 (30.96)		0.305		0.014		2.08	
	Already done this	25 (1.14)		0.013		0.004		2.57	
**Willingness to get digitally tracked and tested for COVID-19 (If these options were available to you, how likely would you be to participate in them?): installing an app on your phone that tracks your location and sends push notifications if you might have been exposed to COVID-19**									
	Extremely likely	283 (12.92)		0.124		0.011		2.21	
	Very likely	307 (14.02)		0.149		0.012		2.47	
	Moderately likely	472 (21.55)		0.217		0.013		2.28	
	Not too likely	386 (17.63)		0.173		0.012		2.34	
	Not likely at all	718 (32.79)		0.325		0.015		2.15	
	Already done this	15 (0.68)		0.007		0.003		2.98	
**Willingness to get digitally tracked and tested for COVID-19 (If these options were available to you, how likely would you be to participate in them?):** **using a website to log your symptoms and location and get recommendations about COVID-19**									
	Extremely likely	229 (10.46)		0.097		0.010		2.37	
	Very likely	315 (14.38)		0.152		0.012		2.37	
	Moderately likely	537 (24.52)		0.232		0.013		2.02	
	Not too likely	432 (19.73)		0.207		0.014		2.48	
	Not likely at all	637 (29.09)		0.292		0.014		2.20	
	Already done this	27 (1.23)		0.012		0.004		2.58	
**Willingness to get digitally tracked and tested for COVID-19 (If these options were available to you, how likely would you be to participate in them?):** **testing you for COVID-19 infection using a Q-tip to swab your cheek or nose**									
	Extremely likely	552 (25.21)		0.227		0.013		2.08	
	Very likely	493 (22.51)		0.248		0.015		2.48	
	Moderately likely	538 (24.57)		0.231		0.013		2.06	
	Not too likely	256 (11.69)		0.136		0.012		2.63	
	Not likely at all	320 (14.61)		0.143		0.011		2.06	
	Already done this	21 (0.96)		0.012		0.004		2.66	
**Willingness to get digitally tracked and tested for COVID-19 (If these options were available to you, how likely would you be to participate in them?):** **testing you for immunity or resistance to COVID-19 by drawing a small amount of blood**									
	Extremely likely	635 (29.00)		0.257		0.014		2.08	
	Very likely	490 (22.37)		0.246		0.014		2.36	
	Moderately likely	478 (21.83)		0.208		0.013		2.19	
	Not too likely	252 (11.51)		0.132		0.012		2.55	
	Not likely at all	314 (14.34)		0.147		0.011		2.21	
	Already done this	8 (0.37)		0.004		0.003		3.14	

^a^Percentages may not sum up to 100 due to rounding. Subcategories may not sum up to 2190 due to missing data. As the focus of this study was on willingness, or likelihood, response option 88 was not included in psychometric analyses. Only 8 (0.37%) participants responded with option 88.

#### Correlates of Willingness to Participate in Pandemic-Related Screening and Tracking

Table 2 details each item that included preventive behaviors conducted in response to COVID-19 for which participants responded to a checklist of items (eg, “worn a face mask,” “worked from home”; “yes” coded 1 and “no” code 0) adapted from the Understanding America Survey [28]. Participants also reported their “frequency of communications with friends and family by phone, text, email, app, or the internet” both (1) in the past month and (2) after COVID-19 began spreading significantly and the public health response began to escalate in the United States in March 2020. Response options range from “1. Basically every day” to “5. Not at all,” and the items were reverse-coded so that higher scores reflected a greater frequency than lower scores. These items were drawn from the Civic Engagement Supplement of the Current Population Survey [29].

#### Sociodemographic Characteristics

The following demographic characteristics were assessed for measurement invariance: age, gender, and race/ethnicity. Participants reported their current age, which the CIS categorized (ie, 18-19, 30-44, 45-59, and ≥60 years) to preserve anonymity, gender (“female” coded 1, “male” coded 0), self-identified race/ethnicity (eg, Black/African American; Hispanic or Latino; White; multiple other races and ethnicities, such as Asian Indian and Native Hawaiian), and education level (ie, no high school diploma or equivalent, high school diploma or equivalent, some college, bachelor’s degree or greater). Participants also reported the geographical region of the United States in which they lived (ie, the Northeast, the Midwest, the South, the West) and the population density of their lived community (ie, rural, suburban, urban).

### Data Analysis Plan

The psychometric properties of 5 items intended to measure the willingness to participate in pandemic-related screening and tracking, including the willingness to use pandemic-related mHealth tools, were evaluated. Descriptive statistics was conducted, and Cronbach α was determined using Stata version 16 [30], and all other psychometric analyses were performed using Mplus version 8 [31]. Model parameters were estimated with these ordinal-scaled items using weighted least squares estimation and Delta parameterization [31]. Model fit was established using 2 of 3 criteria: root mean square error of approximation (RMSEA)≤0.06, comparative fit index (CFI)≥0.95, and standardized root mean square residual (SRMR)≤0.08 [32,33].

Using stage 1 as an exploratory sample, the number of underlying factors assessed by the measure was identified using exploratory factor analysis (EFA). A 1-factor solution was compared against a 2-factor solution, and if the 2-factor solution seemed appropriate, it was compared with a 3-factor solution, and so on. The correct number of factors was determined by evaluating the model fit statistics, eigenvalues (>1), scree plots, and plausibility of emergent factors. Next, using stage 2 as a validation sample, the number of factors was confirmed using CFA. Such measurement models, or latent variables, constructed through CFA account for measurement error. In addition, reliability was assessed using Cronbach α and evaluated convergent and discriminant validity based on associations of the factor(s) with potential correlates of the willingness to participate in pandemic-related screening and tracking. Finally, using stage 3 as a multiple-group, or invariance, sample, the extent to which the measure was invariant across groups by age group was tested (ie, 18-29, 30-44, 45-59, ≥60 years), gender (ie, male, female), race or ethnicity (ie, White, Black, Hispanic, other), education level (ie, high school diploma or equivalent or less, some college, bachelor’s degree or greater), geographical region of the United States, and population density of one’s lived community. For race or ethnicity, the “other” category included 48 (2.19%) non-Hispanic Asians (estimated percentage=3.1%) and 109 (4.98%) individuals of other races or ethnicities (estimated percentage=5.5). The first 2 levels of education, “no high school diploma or equivalent” and “high school diploma or equivalent,” were combined due to the relatively small number of participants (n=98, 4.47%) reporting no high school diploma.

Three levels of measurement invariance were tested: configural, metric, and scalar invariance. A detailed description of conducting measurement invariance analyses with ordinal items is beyond the scope of this paper, although the procedure is briefly described here. First, configural invariance was tested, which is the least strict form of invariance. Specifically, whether each group had the same basic configuration (eg, each group has the same indicators loading onto the same factors in the same direction, positive or negative) was noted. For configural invariance, factor loadings are free to vary across groups, thresholds (ie, the ordinal variable equivalents of intercepts for continuous variables) are free to vary across groups, scale factors are fixed to 1 in all groups, factor means are fixed to 0 for all groups, and factor variances are free to vary across groups [31]. Second, metric invariance was tested, which is the next level of invariance. For metric invariance, factor loadings are constrained to be equal across groups, some thresholds are constrained to be equal across groups, scale factors are fixed to 1 in one group and free to vary in the other groups, and factor means are fixed to 0 in 1 group and free to vary in the other groups [31]. Third, scalar invariance was determined, which indicates strong invariance. For scalar invariance, thresholds are then constrained to be equal across groups. Scalar invariance is the minimum for testing group differences in underlying factor means [34,35]. To compare successive invariance models, a difference in the CFI (ΔCFI)≥0.010 was used to confirm the next level of invariance [34]. Essentially, a lack of worsened model fit with increased constraints indicates measurement invariance.

Given scalar invariance, whether participants differed in the mean levels of the underlying factor by age (reference: age≥60 years), gender (reference: male), race/ethnicity (reference: White), education level (reference: no high school diploma), geographical region of the United States (reference: the Northeast), and population density of one’s lived community (reference: rural) was assessed. To test mean differences, the willingness for screening and tracking, or latent variable, was standardized such that its mean was 0 and SD was 1. The factor mean remained 0 for the reference group and was freely estimated for the other groups. As such, the resulting mean for the nonreference groups reflected the difference in the mean from the reference group in SD units.

To cross-validate the factor analytic findings [36], the factor and invariance analyses (ie, EFA, CFA, measurement invariance testing) and factor mean difference tests in the other stages were repeated. For example, the EFA conducted with the exploratory, stage 1 sample was repeated with the stage 2 and 3 samples.

Given the complex nature of the survey data, the study adjusted for sampling weight, which was the inverse of the probability of selection in the sample. Stratification was also accounted for using pseudostrata based on census tracts; pseudostrata were used to preserve confidentiality. Per the data producer, NORC, cluster variables were not included in the publicly available data sets because of negligible cluster effects (ie, SEs were unaffected). Additionally, excluding these variables further preserved confidentiality. Missing data (up to 13/2190, 0.6%, missing in stage 1; up to 139/2238, 6.2%, missing in stage 2; and up to 45/2047, 2.2%, missing in stage 3 across primary analyses) were handled using listwise deletion, which is typically robust to violations of random missingness and yields appropriate SEs despite a loss of statistical power [37].

## Results

### Descriptive Statistics and Factor Structure

Table 1 displays the sample characteristics in Stage 1. Table 3 shows the results of the EFA. The highest eigenvalue was 3.721, followed by 0.704, 0.236, 0.179, and 0.161. The eigenvalues, along with a review of a scree plot, supported a 1-factor solution. However, as shown in Table 3, the RMSEA, CFI, and SRMR indicated that the 1-factor solution fit the data poorly. As such, the well-fitting 2-factor solution was selected despite the eigenvalue falling short of 1.0 by just under 30%. As displayed in Table 4, 3 items loaded onto the first factor and 2 loaded onto the second factor. Given that 2-item measures are not reliable [38-40], only the first factor was selected. Based on the themes of the factor items, this factor was named “willingness to use pandemic-related mHealth tools.” The EFA using the stage 2 and 3 samples was repeated, and the results were essentially the same (see Multimedia Appendix 1, Table S1).

**Table 3:** EFA^a^ and CFA^b^ of items in a measure of the willingness for pandemic-related screening and tracking.

Measures		EFA (N=2099)			CFA (N=2179)
		1-Factor solution	2-Factor solution	Factor 1 from 2-factor solution^c^	
**Fit index**					
	*χ*^2^ (*df*), *P* value	596.24 (5), <.001	4.08 (1), .04	0 (0), <.001	
	RMSEA^d^ (CI_.90_)	0.237 (0.221-0.254)	0.038 (0.005-0.080)	0 (0-0)	
	CFI^e^	0.953	1.000	1.000	
	SRMR^f^	0.122	0.005	0	

^a^EFA: exploratory factor analysis.

^b^CFA: confirmatory factor analysis.

^c^Factor 1 was fully saturated, as it was a latent variable with 3 indicators. Because it was fully saturated, it had perfect model fit. Factor 2 only had 2 items. As such, factor 2 was underidentified and could not be fit to the data as a separate measurement model.

^d^RMSEA: root mean square error of approximation.

^e^CFI: comparative fit index.

^f^SRMR: standardized root mean square residual.

**Table 4:** Factor loadings.

Item^a^	EFA^b^ 1-factor solution	EFA 2-factor solution		CFA^c^ factor 1 from 2-factor solution^d^
	Factor 1	Factor 1	Factor 2	Factor 1 (willingness for digital tracking)
Installing an app on your phone that asks you questions about your own symptoms and provides recommendations about COVID-19	0.893 (0.009)^e^	1.025 (0.032)^e^	–0.131 (0.041)	1.000 (0)^e^
Installing an app on your phone that tracks your location and sends push notifications if you might have been exposed to COVID-19	0.859 (0.010)^e^	0.869 (0.011)^e^	0.011 (0.005)	0.956 (0.011)^e^
Using a website to log your symptoms and location and get recommendations about COVID-19	0.852 (0.010)^e^	0.827 (0.029)^e^	0.065 (0.035)	0.962 (0.009)^e^
Testing you for COVID-19 infection using a Q-tip to swab your cheek or nose	0.861 (0.012)^e^	0.098 (0.108)	0.831 (0.110)^e^	N/A^f^
Testing you for immunity or resistance to COVID-19 by drawing a small amount of blood	0.849 (0.012)^e^	–0.007 (0.005)	0.919 (0.032)^e^	N/A

^a^For each item, the question was, “There are some options for testing and tracking people who may have COVID-19 in order to help slow the spread of this virus. If these options were available to you, how likely would you be to participate in them?” Response options were as follows: “1. Extremely likely,” “2. Very likely,” “3. Moderately likely,” “4. Not too likely,” “5. Not likely at all,” and “88. Already done this.” As the focus of this study was on willingness, or likelihood, response option 88 was not included in psychometric analyses. Only 8 (0.37%) participants responded with option 88. Possible scores for each item ranged from 1 to 5 and were reverse-coded so that higher scores indicated greater willingness. Unstandardized factor loadings are presented.

^b^EFA: exploratory factor analysis.

^c^CFA: confirmatory factor analysis.

^d^Factor 1 was fully saturated, as it was a latent variable with 3 indicators. Because it was fully saturated, it had perfect model fit. Factor 2 only had 2 items. As such, factor 2 was underidentified and could not be fit to the data as a separate measurement model.

^e^Factor loadings load strongly onto the underlying factor.

^f^N/A: not applicable.

### One-Dimensionality and Reliability

Table 3 shows the results of CFA using the stage 2, or validation, sample. As indicated by the factor loadings, a 1-factor structure characterized the 3 items. The RMSEA, CFI, and SRMR showed perfect fit because the model was just-identified, or fully saturated (ie, *df*=0) with only 3 indicators. In conjunction with EFA, the factor loadings of CFA suggested that the underlying construct was well characterized by the 3 items (Table 4). The CFA was repeated with the stage 1 and 3 samples, and the results were essentially the same (see Multimedia Appendix 1, Table S2). Thus, a one-dimensionality structure was cross-validated across samples in the CIS.

Additionally, the measure showed good reliability in the validation sample (Cronbach α=.90). The measure also showed equivalent reliability in the stage 1 sample (Cronbach α=.90) and the stage 2 sample (Cronbach α=.89).

### Convergent and Discriminant Validity

The measure showed convergent and discriminant validity in its correlations and noncorrelations based on the validation sample (see Table 5). Specifically, the underlying factor of the willingness to use pandemic-related mHealth tools was associated with most variables reflecting protective behaviors taken in response to COVID-19 (eg, “worn a face mask,” “avoided public or crowded places”). Although, the willingness to use pandemic-related mHealth tools was not associated with digital communication (ie, communication via phone, text, email, app, or the internet) with friends and family prior to the spread of COVID-19 in the United States in March 2020, the willingness to use pandemic-related mHealth tools was positively associated with digital communication with friends and family after COVID-19 began spreading. Additionally, the variable was positively associated with the 2 items that were dropped from the measure: willingness to be tested for COVID-19 via a swab in the nose or cheek and willingness to be tested for immunity or resistance to COVID-19 via a small blood draw.

**Table 5:** Construct validity of a measure of the willingness to use mHealth tools for pandemic-related screening and tracking based on correlations with preventive behaviors, digital communication with friends and family before and after the spread of COVID-19 in the United States, and willingness to provide biological specimens for COVID-19–related testing (N=2179).

Behavioral responses to COVID-19	r^a^	SE	*P* value	RMSEA^b^	CFI^c^	SRMR^d^
Canceled a doctor appointment	0.180	0.039	<.001	0.009	1.000	0.006
Worn a face mask	0.313	0.041	<.001	<0.001	1.000	0.002
Visited a doctor or hospital	0.027	0.050	.60	0.022	1.000	0.013
Canceled or postponed work activities	0.207	0.039	<.001	<0.001	1.000	0.005
Canceled or postponed school activities	0.111	0.044	.01	0.010	1.000	0.007
Canceled or postponed dentist or other appointments	0.227	0.037	<.001	0.034	1.000	0.010
Canceled outside housekeepers or caregivers	0.182	0.050	<.001	0.031	1.000	0.014
Avoided some or all restaurants	0.330	0.038	<.001	<0.001	1.000	0.003
Worked from home	0.132	0.039	.001	0.011	1.000	0.007
Studied at home	0.065	0.048	.18	0.011	1.000	0.009
Canceled or postponed pleasure, social, or recreational activities	0.258	0.040	<.001	0.013	1.000	0.006
Stockpiled food or water	0.242	0.039	<.001	0.017	1.000	0.007
Avoided public or crowded places	0.329	0.043	<.001	<0.001	1.000	0.005
Prayed	0.070	0.039	.07	<0.001	1.000	0.005
Avoided contact with high-risk people	0.248	0.038	<.001	<0.001	1.000	0.004
Washed or sanitized hands	0.240	0.54	<.001	<0.001	1.000	0.002
Kept a 6-foot distance from those outside my household/your household	0.282	0.050	<.001	<0.001	1.000	0.001
Stayed home because I felt unwell/you felt unwell	0.207	0.050	<.001	<0.001	1.000	0.011
Wiped packages entering my home/your home	0.297	0.036	<.001	0.016	1.000	0.007
After COVID-19 began spreading in the United States in March 2020: frequency of communications with friends and family by phone, text, email, app, or the internet in the past month	0.110	0.036	.002	<0.001	1.000	0.003
Before COVID-19 began to spread in the United States in March 2020: frequency of communications with friends and family by phone, text, email, app, or the internet in a typical month	0.040	0.035	.26	<0.001	1.000	0.001
Willingness to get tested for COVID-19 infection using a Q-tip to swab your cheek or nose	0.709	0.017	<.001	0.044	0.999	0.005
Willingness to get tested for immunity or resistance to COVID-19 by drawing a small amount of blood	0.684	0.019	<.001	0.022	1.000	0.004

^a^Standardized covariance.

^b^RMSEA: root mean square error of approximation.

^c^CFI: comparative fit index.

^d^SRMR: standardized root mean square residual.

### Measurement Invariance

Tests of measurement invariance by age, gender, race/ethnicity, and education level were conducted. The findings are detailed as next.

#### Measurement Invariance by Age

For age, the configural model had perfect model fit because it was fully saturated (*df*=0). All factor loadings were significant and in the expected direction for each group. Thus, there was configural invariance by age group. The RMSEA, CFI, and SRMR of the more constrained metric model, which had the same configuration (eg, same pattern of size and direction of factor loadings) of the configural model without being fully saturated, also showed good model fit (see Table 6). The measure showed metric invariance (ΔCFI<0.001) and scalar invariance (ΔCFI=0.001).

**Table 6:** Measurement invariance by age group, gender, race/ethnicity, education level, geographical region of the United States, and population density of one’s community of residence for a measure of the willingness to use mHealth tools for pandemic-related screening and tracking.

Fit index			Invariance models						
			Configural		Metric		Scalar		
**Age group^a^ (N=2036)**									
	*χ*^2^ (*df*), *P* value	0.00 (0), <.001		1.52 (6), .96		36.59 (30), .19			
	RMSEA^b^ (CI_.90_)	0 (0-0)		0 (0-0)		0.021 (0-0.041)			
	CFI^c^	1.000		1.000		1.000			
	SRMR^d^	0		0.002		0.019			
**Gender^e^ (N=2036)**									
	*χ*^2^ (*df*), *P* value	0 (0), <.001		8.133 (2), .02		17.79 (10), .06			
	RMSEA (CI_.90_)	0 (0-0)		0.055 (0.020-0.096)		0.028 (0-0.048)			
	CFI	1.000		1.000		0.999			
	SRMR	0		0.006		0.009			
**Race/ethnicity^f^ (N=2001)**									
	*χ*^2^ (*df*), *P* value	0 (0), <.001		4.42 (6), .62		28.14 (30), .56			
	RMSEA (CI_.90_)	0 (0-0)		0 (0-0.049)		0 (0-0.031)			
	CFI	1.000		1.000		1.000			
	SRMR	0		0.004		0.013			
**Education level^g^ (N=2036)**									
	*χ*^2^ (*df*), *P* value	0 (0), <.001		5.49 (4), .24		24.52 (20), .22			
	RMSEA (CI_.90_)	0 (0-0)		0.023 (0-0.066)		0.018 (0-0.040)			
	CFI	1.000		1.000		1.000			
	SRMR	0		0.004		0.013			
**Geographical region of the United States^h^ (N=2036)**									
	*χ*^2^ (*df*), *P* value	0 (0), <.001		6.80 (6), .34		36.66 (30), .19			
	RMSEA (CI_.90_)	0 (0-0)		0.016 (0-0.062)		0.021 (0-0.0410			
	CFI	1.000		1.000		0.999			
	SRMR	0		0.005		0.016			
**Population density of one’s lived community^i^ (N=2036)**									
	*χ*^2^ (*df*), *P* value	0 (0), <.001		1.986 (4), .74		16.63 (20), .68			
	RMSEA (CI_.90_)	0 (0-0)		0 (0-0.041)		0 (0-0.027)			
	CFI	1.000		1.000		1.000			
	SRMR	0		0.002		0.011			

^a^Age groups were 18-29, 30-44, 45-59, and ≥60 years.

^b^RMSEA: root mean square error of approximation.

^c^CFI: comparative fit index.

^d^SRMR: standardized root mean square residual.

^e^Gender categories are male and female.

^f^Race categories were Black/African American, Hispanic/Latino, White, and other race/ethnicity.

^g^Education categories were high school diploma or equivalent or less, some college, and bachelor’s degree or greater.

^h^Geographical regions of the United States were the Northeast, the Midwest, the South, and the West.

^i^The population density of one’s lived community was represented as rural, suburban, and urban.

#### Measurement Invariance by Gender

For gender, the fully saturated configural model showed perfect global fit statistics, but the metric model also fit the data adequately based on the RMSEA, CFI, and SRMR (see Table 6). All factor loadings were significant and in the expected direction for each group. Thus, there was configural invariance by gender. Next, the measure showed metric invariance (ΔCFI<0.001) and scalar invariance (ΔCFI=0.001).

#### Measurement Invariance by Race/Ethnicity

For the race/ethnicity categories, the fully saturated configural model again showed perfect fit. However, the metric model also fit the data well based on the RMSEA, CFI, and SRMR (see Table 6). All factor loadings were significant and in the expected direction for each group. Thus, there was configural invariance by race/ethnicity. Next, the measure showed metric invariance (ΔCFI<0.001) and scalar invariance (ΔCFI<0.001).

#### Measurement Invariance by Education Level

For the education categories, the fully saturated configural model again showed perfect fit. However, the metric model also fit the data well based on the RMSEA, CFI, and SRMR (see Table 6). All factor loadings were significant and in the expected direction for each group. Thus, there was configural invariance by education level. Next, the measure showed metric invariance (ΔCFI<0.001) and scalar invariance (ΔCFI<0.001).

#### Measurement Invariance by Geographical Region of the United States

For the geographical regions of the United States, the fully saturated configural model again showed perfect fit. However, the metric model also fit the data well based on the RMSEA, CFI, and SRMR (see Table 6). All factor loadings were significant and in the expected direction for each group. Thus, there was configural invariance by geographical region. Next, the measure showed metric invariance (ΔCFI<0.001) and scalar invariance (ΔCFI<0.001).

#### Measurement Invariance by Population Density of One’s Lived Community

For the population density of one’s lived community, the fully saturated configural model again showed perfect fit. However, the metric model also fit the data well based on the RMSEA, CFI, and SRMR (see Table 6). All factor loadings were significant and in the expected direction for each group. Thus, there was configural invariance by population density. Next, the measure showed metric invariance (ΔCFI<0.001) and scalar invariance (ΔCFI<0.001).

#### Measurement Invariance in Stage 1 and 2 Samples

The tests of measurement invariance were repeated across all the groupings in the stage 1 and 2 samples. The measure showed measurement invariance in the same way in the stage 1 sample (Multimedia Appendix 1, Table S3) and the stage 2 sample (Multimedia Appendix 1, Table S4) as it did in the stage 3 sample.

### Group Differences in Factor Means

Factor means showed no statistically significant differences by age, gender, education level, or geographical region of the United States, but there were differences by racial/ethnic group and by population density of one’s lived community. Specifically, compared to older adults aged 60 years and more, there were no mean differences in the willingness to use mHealth tools for adults aged 18-29 (ΔM=0.19, SE=0.11, *P*=.10), 30-44 (ΔM=–0.03, SE=0.09, *P*=.97), or 45-49 (ΔM=0.06, SE=0.10, *P*=.56) years. In addition, men and women did not differ (ΔM=0.07, SE=0.06, *P*=.23). Additionally, compared to adults with a high school diploma, its equivalent, or less, there were no differences in adults with some college education (ΔM=–0.17, SE=0.09, *P*=.05) and adults with a bachelor’s degree or greater (ΔM=0.14, SE=0.08, *P*=.09). Adults who lived in the Midwest (ΔM=0.16, SE=0.12, *P*=.17), the South (ΔM=0.08, SE=0.11, *P*=.49), and the West (ΔM=0.04, SE=0.11, *P*=.76) did not differ from adults in the Northeast. However, compared to White Americans, all racial/ethnic minority groups, including Black (ΔM=0.40, SE=0.09, *P*<.001), Hispanic (ΔM=0.31, SE=0.09, *P*=.001), or other (ΔM=0.45, SE=0.12, *P*<.001) Americans showed a greater willingness to use mHealth tools by 31%-45% of a SD unit. Finally, compared to adults who lived in rural areas, adults who lived in suburban (ΔM=–0.30, SE=0.13, *P*=.001) and urban (ΔM=–0.42, SE=0.12, *P*<.001) areas showed lower willingness to use mHealth.

These factor mean difference tests were repeated with the stage 1 and 2 samples. For the stage 1 sample, there were several differences compared to the stage 3 sample. Specifically, there were less consistent differences by racial/ethnic group and no differences by the population density of one’s lived communities. Black Americans did not differ from White Americans in the stage 1 sample (ΔM=0.30, SE=0.22, *P*=.18). Additionally, adults did not differ based on the population density of their lived communities in the stage 1 sample; there were no significant differences between adults who lived in rural areas and adults who lived in suburban (ΔM=0.15, SE=0.21, *P*=.48) or urban (ΔM=–0.03, SE=0.17, *P*=.88) areas.

For the stage 2 sample, there were also several differences compared to the stage 3 multiple-group sample. Specifically, in contrast to the stage 3 sample, there were no differences by racial/ethnic group or the population density of one’s lived community; however, there was a difference by education. White Americans did not differ from Black (ΔM=0.15, SE=0.16, *P*=.36), Hispanic (ΔM=0.20, SE=0.13, *P*=.11), or other (ΔM=0.26, SE=0.18, *P*=.15) Americans in the stage 2 sample. Additionally, adults who lived in suburban areas (ΔM=–0.14, SE=0.13, *P*=.40) and urban (ΔM=–0.16, SE=0.13, *P*=.22) areas did not differ from adults who lived in rural areas in their willingness to use mHealth. However, adults with at least a college degree showed a greater willingness to use mHealth tools than adults with a high school diploma or less (ΔM=0.22, SE=0.11, *P*=.04).

## Discussion

### Principal Findings

Studies that assess the willingness to use mHealth tools often rely on a single item or a collection of ad hoc questions. Validated scales of the willingness to use mHealth tools are rare, possibly nonexistent. Such a measure could be used in population-based surveys, public health surveillance, selection of appropriate samples for mHealth-based intervention development, or screening of patient populations in clinical settings—particularly in times of major pandemics, such as COVID-19. This study psychometrically evaluated such a measure, originally deployed as part of the CIS national probability household survey. The measure initially included 5 items, 3 related to the willingness to use mHealth tools for pandemic-related screening and tracking and 2 about the willingness to provide salivary, mucosal, or blood samples for pandemic-related testing. Ultimately, a 3-item, 1D measure of the willingness to use pandemic-related mHealth tools emerged from these 5 items. Although the variable reflected by the 3-item measure was highly correlated with participants’ reported willingness to provide biological specimens for testing, the 3 items measure a unique construct distinct from the items about providing biological specimens. Notably, the measure showed invariance across groups by age, gender, race/ethnicity, education level, geographical region of the United States, and population density of one’s lived community, indicating that it measured the same construct in the same way across demographic and cultural groups and geographical representations. The factor analytic psychometric findings were duplicated across all 3 samples, which bolstered the conclusions about the psychometric fitness of the 3-item measure. Thus, the measure can be administered to diverse groups and be used to test differences between groups in their willingness to use mHealth tools.

In the 3-item measure of the willingness to use mHealth tools, 2 items asked about participants’ willingness to download a mobile app and 1 item asked about participants’ willingness to use a website to track symptoms and possible exposures and get recommendations. A prior study evaluating a web browser–based app intended to be compatible across different smartphone operating systems (eg, Android vs iOS) found that many participants would have preferred a native app that would presumably require a download [41,42]. This study suggests that regardless of user preference for a browser-based versus a native app, the items collectively assessed an underlying construct of the willingness to use mHealth tools, broadly.

Higher scores on the measure of the willingness to use mHealth tools were associated in expected ways with other variables, including the variables reflected by the items of the willingness to provide biological specimens for pandemic-related screening and tracking. This empirical link is consistent with the available literature. For example, a US study found that most internet-using participants reported a willingness to use at-home collection methods to provide biological specimens for COVID-19 research [3]. Another US study showed that participants who self-collected biological specimens via throat swabs and dried blood spots during a telehealth session found the procedure acceptable [5]. Telehealth sessions often occur via a mobile app on one’s phone or another portable device.

Additionally, higher scores on the measure of the willingness to use mHealth tools were positively correlated with participants having engaged in COVID-19–preventive behaviors, such as wearing masks or maintaining a 6-foot distance from people outside of one’s household. Thus, the measure tracks with other items that show a willingness to participate in the public health response to stem the pandemic, while still retaining its unique quality as a measure that assesses the willingness to use pandemic-related technological tools.

Participants who scored higher in the willingness to use mHealth tools for pandemic-related screening and tracking communicated more with friends and family via phone, text, email, app, or the internet after COVID-19 began to spread in the United States than participants who scored lower in willing to use mHealth tools. However, the willingness to use mHealth tools for pandemic-related screening and tracking was not associated with communication with friends and family via phone, text, email, app, or the internet before the spread of COVID-19 in the United States. These associations further support an underlying construct of the measure that specifically assesses an adaptation to using digital tools in response to a pandemic.

Participants did not differ in the willingness to use mHealth tools for pandemic-related screening and tracking by age or gender, which is consistent with a previous study that used a 1-item measure of the willingness to participate in contact tracing via a mobile app among users of the National Health Service in the United Kingdom [13]. There were also no differences by education level in this study. In addition, in this analyses, White participants showed less willingness to use mHealth tools than racial/ethnic minority participants. Previous research has shown no consistent findings indicating that White participants are less willing to engaging in COVID-19–preventive behaviors, such as mHealth tools, than other races. However, some variability in demographics may be accounted for by other factors, such as political ideology. For example, in the United States, conservative political ideology or partisanship are associated with a low likelihood of COVID-19–preventive behaviors [43], including mask usage [44] and vaccine trust [45]. Although there is typically quite a bit of heterogeneity within racial and ethnic groups and political parties with respect to political ideology, White Americans lean more toward affiliating with the Republican Party than the Democratic Party, a large majority of African Americans are more likely to affiliate with the Democratic Party than the Republican Party, and Hispanic and Asian Americans lie in between [46,47].

In terms of geographical differences, although there were no detectable differences based on the geographical region of the United States in which participants were located, participants differed based on whether they lived in a rural, suburban, or urban area. Specifically, adults who lived in rural areas showed a greater willingness to use mHealth tools for public health screening and tracking than adults who lived in suburban or urban areas. These findings are consistent with the prior literature on mHealth for rural populations. For example, a recent study of the association between access to mental health counseling and interest in rural telehealth found that although rural residents have less access to mental health counseling and the internet than urban residents, rural residents have more interest in telehealth [48]. Additionally, the less access participants had to mental health counseling, the greater their interest in telehealth [48]. Thus, a lack of access to in-person COVID-19–related testing and services or public health infrastructure to disseminate information in rural areas may coincide with the greater interest in the use of pandemic-related mHealth tools observed in this study. However, there are disparities in telehealth usage, as people who live in communities with limited broadband coverage, such as many rural areas, are less likely to use telehealth [49]. As such, it could be useful to assess willingness separately from actual use.

When tests of mean differences were repeated in the 2 earlier CIS samples, there tended to be fewer group differences in the earlier stage 1 and 2 samples than in the later state 3 sample. For example, in stage 1 of data collection in late April 2020, there were no significant differences by race or ethnicity in the willingness to use mHealth tools. However, by stage 3 of data collection in late May and early June 2020, racial and ethnic minority adults showed a greater willingness to use mHealth tools compared to White adults. Additionally, rural residents did not show a significant difference in the willingness to use pandemic-related mHealth tools compared to suburban or urban residents until the last instance of CIS data collection in late May and early June 2020, several months into the public health response to the COVID-19 pandemic. The differences in interest may have been due to changes in public perceptions of the need for COVID-19–related information and services among adults of color and rural adults as more information emerged about COVID-19.

Even though the CIS was cross-sectional in nature with no longitudinal tracking of specific households, the differences in the statistical significance of group mean differences across stages of data collection likely reflect the changes in the public’s willingness to use mHealth tools over time. Specifically, the CIS was intended to capture cross sections of attitudes and behaviors across the United States in ways that are highly representative of various sociodemographic groups at the time of data collection. Thus, each successive stage of data collection could be interpreted in terms of changes in American attitudes over time. Given how quickly information about COVID-19 and appropriate preventive responses evolved in the early months of the pandemic, extant studies may help to contextualize this study’s findings. For example, in a US study, adults with lower health literacy had greater confidence in the federal government response [50]. Thus, as information about the COVID-19 pandemic rapidly evolved, groups with greater representation of adults with low health literacy might have shown a greater willingness to use mHealth tools. Additionally, a study of Australian adults found that those who viewed themselves as being at intermediate or high risk due to COVID-19 were concerned about having to self-isolate if diagnosed with COVID-19 and those who perceived COVID-19 as a severe condition were more likely to engage in preventive behaviors than adults who did not view themselves as being at risk, have concerns about self-isolation, or perceive COVID-19 as severe [51]. Such concerns could explain variations in group differences in the willingness to use mHealth tools over time for people of color and rural residents in this study after a few months had elapsed early in the pandemic.

The 3-item CIS measure has broad applicability across different use cases. For example, the measure can be used when predicting who would be willing to use mHealth tools before rolling out an mHealth intervention or testing whether those who score higher in the willingness to use mHealth tools give higher usability scores on or engage in more extensive use of a specific app than people who score lower on the willingness to use mHealth tools. Other examples might include whether an intervention to increase the willingness or intention to use mHealth tools is effective or whether using a specific mobile app increases the general willingness to use mHealth tools. Another use case is to determine whether populations that are adversely affected by digital health inequities show different levels of the willingness to use digital health tools than those who are not affected by digital health inequities. For instance, patients with relatively low access to internet-enabled technology or broadband might be willing to use such tools despite low apparent uptake, which has been demonstrated in prior research [21].

### Limitations

Although this study has many strengths, there were several notable limitations. Specifically, the study was conducted in cross-sectional stages. Thus, no temporal or causal conclusions can be drawn. Additionally, the timing of data collection for the CIS within the first few months of the public health response to the pandemic may also be a limitation. Specifically, testing was not yet widespread, and although there was a large proliferation of contact-tracing apps within the first few months of the pandemic, the number of apps reached its zenith about 2 months after the final CIS data collection [8]. Thus, there may have been a relatively small number of widely available mHealth tools for symptom assessment and contact tracing at the time of CIS data collection, particularly in the early stages of data collection. Some of the differences in the willingness to use mHealth tools might have varied with increased proliferation of testing and public health tools. Finally, any other psychometrically tested measures of the willingness to use mHealth tools for screening and tracking could not be identified. Thus, there is limited research available against which to compare this study’s measure.

### Conclusion

In conclusion, the study findings have research and applied implications. Broadly, more population-level studies are needed to examine the willingness to use mHealth tools in response to public health issues, including pandemics. The measure can facilitate these efforts. Additionally, researchers have argued that the use of mHealth tools should be combined with at-home specimen collection methods to confirm COVID-19 with laboratory analysis, as symptom-based screening alone may be insufficient to serve as a leading indicator of new COVID-19 cases or even determine who should be tested [52]. Additionally, the CIS measure asked about the willingness to download a mobile app voluntarily. However, apps can also be automatically downloaded such that prospective users must opt out. Studies could adapt the CIS measure or test additional items based on whether participants would be willing to keep an automatically downloaded app. However, given that some may oppose digital health measures due to concerns about their rights and privacy [15,16,53-57], assessing the willingness to use automatically downloaded opt-out apps should be assessed separately and compared against the willingness to use apps that require users to download. This study focused on voluntary access to digital health tools, which would likely be the most common scenario, and a prior study did not find marked differences in the willingness to use user-downloaded apps versus automatically downloaded apps [15].

In addition, studies have found that people prefer at-home specimen collection methods over going to a drive-through or clinic [3,4]. Thus, the measure can be used to screen or measure peoples’ willingness to use mHealth tools as part of a broader screening and tracking approach that combines at-home self-collection of biological specimens to control a pandemic or outbreak. The measure of the willingness to use pandemic-related mHealth tools can also be used in mHealth and pandemic-related research to screen participants for low, moderate, or high willingness to use mHealth tools. The measure can also be used in studies to develop interventions to enhance the use of mHealth tools. Additionally, in applied settings, clinicians and other professionals can use the measure as a brief screener to determine, for example, how much of their patient population would be open to using mHealth tools.

## Appendix

Multimedia Appendix 1 Supplementary Tables S1-S4.

## Abbreviations

CFA: confirmatory factor analysis
CFI: comparative fit index
CIS: COVID Impact Survey
EFA: exploratory factor analysis
mHealth: mobile health
NORC: National Opinion Research Center
RMSEA: root mean square error of approximation
SRMR: standardized root mean square residual

## Data Availability

The data for this paper were collected by the National Opinion Research Center (NORC) at the University of Chicago and made publicly available by the funder, the Data Foundation. The data can be accessed in Ref. [24].
