# Supplementary material for: Developing and Evaluating a Measure of the Willingness to Use Pandemic-Related mHealth Tools Using National Probability Samples in the United States: Quantitative Psychometric Analyses and Tests of Sociodemographic Group Differences
Source: JMIR Form Res. 2023 Feb 7;7:e38298. doi: 10.2196/38298 (PMC9944142; doi:10.2196/38298)
Supplement: Multimedia Appendix 1 [file formative_v7i1e38298_app1.docx]

**Supplementary Table 1. Exploratory analyses of items in a measure of willingness for pandemic-related screening and tracking using Stage 2 and Stage 3 samples^a^**

|  |  | Stag 2 Sample | | | Stage 3 Sample | | |
| --- | --- | --- | --- | --- | --- | --- | --- |
|  |  | EFA (*N*=2,142) | | | EFA (*N*=1,918) | | |
|  |  | 1-Factor Solution | 2-Factor Solution | | 1-Factor Solution | 2-Factor Solution | |
| Fit Index | |  |  | |  |  | |
|  | χ^2^(degrees of freedom), *p*-value | 545.92 (5), *P*<.001 | 1.532 (1), *P*=.22 | | 561.78 (5), *P*<.001 | 1.840 (1), *P*=.18 | |
|  | RMSEA (CI_.90_) | 0.225 (0.209, 0.241) | 0.016 (0.000, 0.062) | | 0.241 (0.224, 0.258) | 0.021 (0.000, 0.069) | |
|  | CFI | 0.974 | 1.00 | | 0.965 | 1.00 | |
|  | SRMR | 0.128 | 0.002 | | 0.122 | 0.003 | |
| Item | | F1 | F1 | F2 | F1 | F1 | F2 |
| 1 | Installing an app on your phone that asks you questions about your own symptoms and provides recommendations about COVID-19 | **0.900 (0.007)** | **0.995 (0.022)** | -0.037 (0.027) | **0.899 (0.007)** | **0.963 (0.028)** | -0.033 (0.036) |
| 2 | Installing an app on your phone that tracks your location and sends push notifications if you might have been exposed to COVID-19 | **0.867 (0.009)** | **0.889 (0.009)** | 0.004 (0.002) | **0.852 (0.010)** | **0.870 (0.010)** | 0.006 (0.003) |
| 3 | Using a website to log your symptoms and location and get recommendations about COVID-19 | **0.871 (0.008)** | **0.868 (0.022)** | 0.039 (0.029) | **0.864 (0.010)** | **0.817 (0.028)** | 0.087 (0.036) |
| 4 | Testing you for COVID-19 infection using a Q-tip to swab your cheek or nose | **0.894 (0.008)** | -0.003 (0.004) | **1.044 (0.125)** | **0.887 (0.008)** | 0.158 (0.131) | **0.800 (0.136)** |
| 5 | Testing you for immunity or resistance to COVID-19 by drawing a small amount of blood | **0.900 (0.008)** | 0.217 (0.216) | **0.686 (0.232)** | **0.863 (0.011)** | -0.007 (0.006) | **0.932 (0.051)** |

|  |  | Stag 2 Sample | | | Stage 3 Sample | | |
| --- | --- | --- | --- | --- | --- | --- | --- |
|  |  | EFA (*N*=2,142) | | | EFA (*N*=1,918) | | |
|  |  | 1-Factor Solution | 2-Factor Solution | | 1-Factor Solution | 2-Factor Solution | |
| Fit Index | |  |  | |  |  | |
|  | χ^2^(degrees of freedom), *p*-value | 545.92 (5), *P*<.001 | 1.532 (1), *P*=.22 | | 561.78 (5), *P*<.001 | 1.840 (1), *P*=.18 | |
|  | RMSEA (CI_.90_) | 0.225 (0.209, 0.241) | 0.016 (0.000, 0.062) | | 0.241 (0.224, 0.258) | 0.021 (0.000, 0.069) | |
|  | CFI | 0.974 | 1.00 | | 0.965 | 1.00 | |
|  | SRMR | 0.128 | 0.002 | | 0.122 | 0.003 | |
| Item | | F1 | F1 | F2 | F1 | F1 | F2 |
| 1 | Installing an app on your phone that asks you questions about your own symptoms and provides recommendations about COVID-19 | **0.900 (0.007)** | **0.995 (0.022)** | -0.037 (0.027) | **0.899 (0.007)** | **0.963 (0.028)** | -0.033 (0.036) |
| 2 | Installing an app on your phone that tracks your location and sends push notifications if you might have been exposed to COVID-19 | **0.867 (0.009)** | **0.889 (0.009)** | 0.004 (0.002) | **0.852 (0.010)** | **0.870 (0.010)** | 0.006 (0.003) |
| 3 | Using a website to log your symptoms and location and get recommendations about COVID-19 | **0.871 (0.008)** | **0.868 (0.022)** | 0.039 (0.029) | **0.864 (0.010)** | **0.817 (0.028)** | 0.087 (0.036) |
| 4 | Testing you for COVID-19 infection using a Q-tip to swab your cheek or nose | **0.894 (0.008)** | -0.003 (0.004) | **1.044 (0.125)** | **0.887 (0.008)** | 0.158 (0.131) | **0.800 (0.136)** |
| 5 | Testing you for immunity or resistance to COVID-19 by drawing a small amount of blood | **0.900 (0.008)** | 0.217 (0.216) | **0.686 (0.232)** | **0.863 (0.011)** | -0.007 (0.006) | **0.932 (0.051)** |

Note. For each item, the question was, “There are some options for testing and tracking people who may have COVID-19 in order to help slow the spread of this virus. If these options were available to you, how likely would you be to participate in them?” Response options were as follows: “1. Extremely likely,” “2. Very likely,” “3. Moderately likely,” “4. Not too likely,” “5. Not likely at all,” and “88. Already done this.” As the focus of the present study was on willingness, or likelihood, we did not include response option 88 in psychometric analyses. Few participants, only eight (0.37%) in the Stage 1 sample, responded with option 88. Possible scores for each item ranged from 1–5 and were reverse-coded so that higher scores indicated greater willingness. Unstandardized factor loadings are presented. Factor loadings in **bold font** load strongly onto the underlying factor. F1=factor 1. F2=factor 2.
**^a^** This table supplements Table 2 in the main text, which reports the results of the exploratory factor analysis with the exploratory Stage 1 sample.

**Supplementary Table 2. Confirmatory analyses of items in a measure of willingness for pandemic-related screening and tracking using Stage 1 and Stage 3 samples^a^**

|  |  | Stage 1 Sample | Stage 3 Sample |
| --- | --- | --- | --- |
|  |  | CFA (*N*=2,125) | CFA (*N*=1,981) |
|  |  | Factor 1 from 2-Factor Exploratory Factor Analysis Solution | Factor 1 from 2-Factor Exploratory Factor Analysis Solution |
| Fit Index | |  |  |
|  | χ^2^(degrees of freedom), *p*-value | 0.000 (0), *P*<.001 | 0.000 (0), *P*<.001 |
|  | RMSEA (CI_.90_) | 0.000 (0.000, 0.000) | 0.000 (0.000, 0.000) |
|  | CFI | 1.000 | 1.000 |
|  | SRMR | 0.000 | 0.000 |
| Item | |  |  |
| 1 | Installing an app on your phone that asks you questions about your own symptoms and provides recommendations about COVID-19 | **1.000 (0.000)** | **1.000 (0.000)** |
| 2 | Installing an app on your phone that tracks your location and sends push notifications if you might have been exposed to COVID-19 | **0.947 (0.012)** | **0.935 (0.011)** |
| 3 | Using a website to log your symptoms and location and get recommendations about COVID-19 | **0.939 (0.0012)** | **0.933 (0.012)** |
| 4 | Testing you for COVID-19 infection using a Q-tip to swab your cheek or nose | --- | --- |
| 5 | Testing you for immunity or resistance to COVID-19 by drawing a small amount of blood | --- | --- |

Note. The three-item factor fully saturated, as it is a latent variable with three indicators. Because it is fully saturated, it has perfect model fit. For each item, the question was, “There are some options for testing and tracking people who may have COVID-19 in order to help slow the spread of this virus. If these options were available to you, how likely would you be to participate in them?” Response options were as follows: “1. Extremely likely,” “2. Very likely,” “3. Moderately likely,” “4. Not too likely,” “5. Not likely at all,” and “88. Already done this.” As the focus of the present study was on willingness, or likelihood, we did not include response option 88 in psychometric analyses. Few participants, only eight participants (0.37%) in the Stage 1 sample, responded with option 88. Possible scores for each item ranged from 1–5 and were reverse-coded so that higher scores indicated greater willingness. Unstandardized factor loadings are presented. Factor loadings in **bold font** load strongly onto the underlying factor.

**^a^** This table supplements Table 2 in the main text, which reports the results of the confirmatory factor analysis with the Stage 2, or validation, sample.

**Supplementary Table 3. Measurement invariance by age category, gender, race/ethnicity, education level, geographical region of the United States, and population density of one’s community of residence for a measure of willingness to use mHealth tools for pandemic-related screening and tracking in the Stage 1 sample^a^**

|  | Invariance Models | | |
| --- | --- | --- | --- |
| Fit Index | Configural | Metric | Scalar |
|  | Age Category (*N*=1,088) | | |
| χ^2^(degrees of freedom), *p*-value | 0.00 (0), *P<*.001 | 10.83 (6), *P*=.09 | 30.97 (30), *P*=.42 |
| RMSEA (CI_.90_) | 0.000 (0.000, 0.000) | 0.054 (0.000, 0.105) | 0.011 (0.000, 0.048) |
| CFI | 1.000 | 0.999 | 1.000 |
| SRMR | 0.000 | 0.009 | 0.021 |
|  | Gender (*N*=1,088) | | |
| χ^2^(degrees of freedom), *p*-value | 0.00 (0), *P<*.001 | 1.05 (2), *P*=.59 | 14.32 (10), *P*=.16 |
| RMSEA (CI_.90_) | 0.000 (0.000, 0.000) | 0.000 (0.020, 0.070) | 0.028 (0.000, 0.058) |
| CFI | 1.000 | 1.000 | 0.999 |
| SRMR | 0.000 | 0.003 | 0.016 |
|  | Race/Ethnicity (*N*=1,088) | | |
| χ^2^(degrees of freedom), *p*-value | 0.00 (0), *P<*.001 | 7.62 (6), *P*=.27 | 39.78 (30), *P*=.11 |
| RMSEA (CI_.90_) | 0.000 (0.000, 0.000) | 0.000 (0.000, 0.089) | 0.035 (0.000, 0.061) |
| CFI | 1.000 | 1.000 | 0.999 |
| SRMR | 0.000 | 0.010 | 0.027 |
|  | Education Level (*N*=1,088) | | |
| χ^2^(degrees of freedom), *p*-value | 0.00 (0), *P<*.001 | 9.52 (4), *P*=.049 | 30.27 (20), *P*=.07 |
| RMSEA (CI_.90_) | 0.000 (0.000, 0.000) | 0.062 (0.003, 0.113) | 0.038 (0.000, 0.063) |
| CFI | 1.000 | 0.999 | 0.998 |
| SRMR | 0.000 | 0.008 | 0.018 |
|  | Geographical Region of the United States (*N*=1,088) | | |
| χ^2^(degrees of freedom), *p*-value | 0.00 (0), *P<*.001 | 6.81 (6), *P*=0.34 | 34.42 (30), *P*=.26 |
| RMSEA (CI_.90_) | 0.000 (0.000, 0.000) | 0.022 (0.000, 0.084) | 0.023 (0.000, 0.053) |
| CFI | 1.000 | 1.000 | 0.999 |
| SRMR | 0.000 | 0.007 | 0.028 |
|  | Population Density of One’s Lived Community (*N*=1,086) | | |
| χ^2^(degrees of freedom), *p*-value | 0.00 (0), *P<*.001 | 4.46 (4), *P*=.35 | 23.74 (20), *P*=.25 |
| RMSEA (CI_.90_) | 0.000 (0.000, 0.000) | 0.018 (0.000, 0.083) | 0.023 (0.000, 0.053) |
| CFI | 1.000 | 1.000 | 0.999 |
| SRMR | 0.000 | 0.006 | 0.018 |

*Note.* Age categories are 18-29, 30-44, 45-59, and 60+ years old. Gender categories are male and female. Race categories are Black/African American, Hispanic/Latino, White, and other race/ethnicity. Education categories are high school diploma or equivalent or less, some college, and bachelor’s degree or greater. Geographical regions of the United States were the Northeast, the Midwest, the South, and the West. The population density of one’s lived community was represented as rural, suburban, and urban.

**^a^** This table supplements Table 4 in the main text, which reports the results of the tests of measurement invariance with the Stage 3, or multiple-group, sample.

**Supplementary Table 4. Measurement invariance by age category, gender, race/ethnicity, education level, geographical region of the United States, and population density of one’s community of residence for a measure of willingness to use mHealth tools for pandemic-related screening and tracking in the Stage 2 sample^a^**

|  | Invariance Models | | |
| --- | --- | --- | --- |
| Fit Index | Configural | Metric | Scalar |
|  | Age Category (*N*=1,111) | | |
| χ^2^(degrees of freedom), *p*-value | 0.00 (0), *P<*.001 | 9.78 (6), *P*=.13 | 34.71 (30), *P*=.25 |
| RMSEA (CI_.90_) | 0.000 (0.000, 0.000) | 0.048 (0.000, 0.100) | 0.024 (0.000, 0.053) |
| CFI | 1.000 | 0.999 | 0.999 |
| SRMR | 0.000 | 0.009 | 0.020 |
|  | Gender (*N*=1,111) | | |
| χ^2^(degrees of freedom), *p*-value | 0.00 (0), *P<*.001 | 0.72 (2), *P*=.70 | 8.30 (10), *P*=.60 |
| RMSEA (CI_.90_) | 0.000 (0.000, 0.000) | 0.000 (0.000, 0.062) | 0.000 (0.000, 0.040) |
| CFI | 1.000 | 1.000 | 1.000 |
| SRMR | 0.000 | 0.002 | 0.011 |
|  | Race/Ethnicity (*N*=1,111) | | |
| χ^2^(degrees of freedom), *p*-value | 0.00 (0), *P<*.001 | 6.81 (6), *P*=.34 | 38.24 (30), *P*=.14 |
| RMSEA (CI_.90_) | 0.000 (0.000, 0.000) | 0.022 (0.000, 0.083) | 0.031 (0.000, 0.058) |
| CFI | 1.000 | 1.000 | 0.999 |
| SRMR | 0.000 | 0.009 | 0.024 |
|  | Education Level (*N*=1,111) | | |
| χ^2^(degrees of freedom), *p*-value | 0.00 (0), *P<*.001 | 2.18 (4), *P*=.70 | 29.29 (20), *P*=.08 |
| RMSEA (CI_.90_) | 0.000 (0.000, 0.000) | 0.000 (0.000, 0.059) | 0.035 (0.000, 0.061) |
| CFI | 1.000 | 1.000 | 0.999 |
| SRMR | 0.000 | 0.004 | 0.019 |
|  | Geographical Region of the United States (*N*=1,111) | | |
| χ^2^(degrees of freedom), *p*-value | 0.00 (0), *P<*.001 | 8.38 (6), *P*=.21 | 40.87 (30), *P*=.09 |
| RMSEA (CI_.90_) | 0.000 (0.000, 0.000) | 0.038 (0.000, 0.092) | 0.036 (0.000, 0.062) |
| CFI | 1.000 | 1.000 | 0.999 |
| SRMR | 0.000 | 0.007 | 0.021 |
|  | Population Density of One’s Lived Community (*N*=1,111) | | |
| χ^2^(degrees of freedom), *p*-value | 0.00 (0), *P<*.001 | 3.32 (4), *P*=.51 | 23.08 (20), *P*=.28 |
| RMSEA (CI_.90_) | 0.000 (0.000, 0.000) | 0.000 (0.000, 0.072) | 0.020 (0.000, 0.051) |
| CFI | 1.000 | 1.000 | 1.000 |
| SRMR | 0.000 | 0.004 | 0.019 |

*Note.* Age categories are 18-29, 30-44, 45-59, and 60+ years old. Gender categories are male and female. Race categories are Black/African American, Hispanic/Latino, White, and other race/ethnicity. Education categories are high school diploma or equivalent or less, some college, and bachelor’s degree or greater. Geographical regions of the United States were the Northeast, the Midwest, the South, and the West. The population density of one’s lived community was represented as rural, suburban, and urban.

**^a^** This table supplements Table 4 in the main text, which reports the results of the tests of measurement invariance with the Stage 3, or multiple-group, sample.
